# 

**Published:** 1923-06

**Authors:** 


### RECENT APPOINTMENTS.

### Medical Officers, Chaplains, etc.

Caley, Frederick Goodman, A.R.C.S., L.R.C.P., Deputy M.O.H., Wandsworth ; M.O.H., Wandsworth.

Clegg, Sydney J., M.D., D.P.H., Deputy M.O.H., Newcastle-on-Tyne ; M.O.H., Corporation of Durban, Natal.

Cohen, Julius ; House Surgeon, St. Mary's Hospital Paddington.

Coulthard, Harold S.,:M.D., D.P.H., House Surgeon, Royal Samaritan Hospital, Glasgow : Assistant M.O. Cardiff.

Evans, W. Glyn, M.D., D.P.H. ; Acting M.O.H., Wrexham Rural District Council.

Ferguson, May Clement, M.B.; Assistant School M.O., Norfolk Education Committee.

Graham, William, M.B., B.S. ; Assistant M.O. Holgate Hospital.

Robertson, James, M.B.D.P.H., Assistant M.O., Blackburn ; M.O., Darwen.

Sanger, Frederick, M.R.C.S., L.R.C.P. ; M.O., Solihull Union.

Stott, W. M. ; M.O., Lowestoft.

Sutton, Surgeon-Captain Edward, C.M.G. , Second-in-Charge, Haslar Hospital.

Tate, John, M.R.C.S., L.R.C.P. ; M.O.H. for Middlesex, £1,400.

Wear, A. H., M.B., Deputy M.O.H. and Tuberculosis M.O., South Shields ; M.O.H., Blackwell and Skegly Rural District Council (Durban University).

### Public Health and Hospital Appointments.

Carleton, Hugh Hadfield, Hon. Assistant Physican, Bristol Hospital.

Gray, Sir Henry, C.M.G., K.B.E., F.R.C.S. ; Chief of the Surgical Staff of the Royal Victoria Hospital, Montreal (Aberdeen University).

Harris, J. H., M.O. Dartmouth Infant Welfare Centre.

Hearn, D. Almoner, Royal Infirmary, Bristol.

Heyer, George, Secretary of the King's College Hospital Medical School : Appeal Secretary, King's College Hospital.

Hulme, Marjorie Alice ; Health Visitor, Warrington County Borough (Royal Liverpool Children's Hospital and York Maternity Hospital).

Hutchinson, Eleanor K., Matron, Paignton and District Hospital (Stanley Hospital, Liverpool).

Jenkins, E. A., C.M.B. ; Health Visitor, Carmarthen County Borough (Maternity Hospital, Newport, and Newport Infirmary).

McCarthy, Maud Jane, Matron-in-Chief Territorial Army Nursing Service ; War Office Representative on the Central Joint Voluntary Aid Detachment Council.

Parsons, M. E., Matron, Victoria Hospital, Southend-on-Sea ; Matron, Huddersfield Royal Infirmary (St. Thomas's Hospital).

Rennison, A. A. A., R.R.C., Matron, Belvedere Hospital Glasgow ; Matron, Ashington Hospital, Northumberland (Portsmouth Hospital).

Robertson, Dorothea, B.A. ; Teacher of Occupational Therapy, Royal Mental Hospital, Glasgow (Cambridge).

Ross, A. I. Almoner, East London Hospital for Children, Shadwell.

Thick, F. J., Sanitary Inspector, Teignmouth Urban District Council ; Sanitary Inspector Paignton Urban District Council. (£244.)

Thomson, Miss, C. Almoner, Norfolk and Norwich Hospital, Norwich.

Trew, B. M., Sanitary Inspector, Horle Urban District Council ; Surveyor and Sanitary Inspector, Chester Rural District Council.

### PUBLIC HEALTH IN PARLIAMENT.

The following are selected as being of general interest from answers recently given by the Minister of Health to questions in Parliament.

### VOLUNTARY HOSPITALS.

Distributions by the Commission.—I understand that up to the present the Commission have distributed £421,033. The Commission have not yet made any recommendation as to the necessity for any future grant or for the continuance of some central body to act as a link between the Local Voluntary Hospital Committees.

### HOUSING.

Non-Parlour Houses (Cost).—For the purpose of calculation, the average cost of a non-parlour house has been estimated at figures varying between £300 and £345, but I hope that the actual cost of building will prove to be below these figures.

Statistics.—According to the survey made by local authorities in England and Wales at the end of 1919 the gross estimate of shortage of working-class houses due to overcrowding was 506,700. But in view of the difficulty of obtaining reliable estimates the Hon. Member must not take it that I regard this as representing the present effective demand for houses.

Slum Clearances.—(Grant in Aid).—The estimate of £230,000 referred to in the memorandum explaining the financial provisions of the Housing Bill was the best estimate that could be made of half the annual deficit on schemes to be approved under clause 1 (3) of the Bill which Local Authorities might be expected to carry out in the near future. If Local Authorities submit schemes which would involve in the aggregate a larger contribution from the Exchequer the matter will be further reviewed on the annual estimates.

### NATIONAL HEALTH INSURANCE.

Approved Societies (Cost of Administration).—I propose to lay before Parliament at an early date the draft of a Regulation to reduce the maximum allowance to Societies for purposes of administration from the present figure of 4s. 10d. to 4s. 5d. per insured member per annum as from January 1, 1924.

Payments to Insurance Practitioners.—In reporting on the Financial Provisions of the National Health Insurance Bill of 1922, the Government Actuary merely indicated that his opinion that the charge proposed by the Bill could be safely borne applied strictly to the proposals of that measure, and should not be held to imply that larger or more protracted additional charges could be borne by Societies. No further report or advice has since been received from him on the subject.

Medical Benefit for Insured Persons over 65.—Careful consideration will be given to the question of the inclusion in the next Bill to amend the National Health Insurance Acts of a clause to enable insured persons who cease employment between the ages of 65 and 70 to remain entitled to Medical Benefit on payment of a weekly contribution sufficient to cover the cost of that benefit.

Medical System.—As a result of discussion with the Insurance Consultative Council, it is not now contemplated that any general rearrangement of the insurance medical system will be made. Certain modifications of the terms of services have been recommended by the Council, but these will not involve any substantial change in the present system.

Insurance Practitioners (Possible Refusal to Do Panel Work.)—Negotiations with representatives of insurance practitioners with a view to the revision of their terms of service have now begun, and the prospects of a successful issue would not be enhanced by any statement at this stage as to the steps which the Government might find it necessary to take in a contingency which I have every reason to hope will not arise.

Women Medical Practitioners.—The total number of women medical practitioners engaged in insurance work in England and Wales is 215.

